# Short-term depression shapes information transmission in a constitutively active GABAergic synapse

**DOI:** 10.1038/s41598-019-54607-y

**Published:** 2019-12-02

**Authors:** Hagar Lavian, Alon Korngreen

**Affiliations:** 1 The Leslie and Susan Gonda Interdisciplinary Brain Research Center, Bar Ilan University, Ramat Gan, 5290002 Israel; 2 The Mina and Everard Goodman Faculty of Life Sciences, Bar Ilan University, Ramat Gan, 5290002 Israel

**Keywords:** Cellular neuroscience, Biophysical models

## Abstract

Short-term depression is a low-pass filter of synaptic information, reducing synaptic information transfer at high presynaptic firing frequencies. Consequently, during elevated presynaptic firing, little information passes to the postsynaptic neuron. However, many neurons fire at relatively high frequencies all the time. Does depression silence their synapses? We tested this apparent contradiction in the indirect pathway of the basal ganglia. Using numerical modeling and whole-cell recordings from single entopeduncular nucleus (EP) neurons in rat brain slices, we investigated how different firing rates of globus pallidus (GP) neurons affect information transmission to the EP. Whole-cell recordings showed significant variability in steady-state depression, which decreased as stimulation frequency increased. Modeling predicted that this variability would translate into different postsynaptic noise levels during constitutive presynaptic activity. Our simulations further predicted that individual GP-EP synapses mediate gain control. However, when we consider the integration of multiple inputs, the broad range of GP firing rates would enable different modes of information transmission. Finally, we predict that changes in dopamine levels can shift the action of GP neurons from rate coding to gain modulation. Our results thus demonstrate how short-term depression shapes information transmission in the basal ganglia in particular and via GABAergic synapses in general.

## Introduction

Short-term depression plays a vital role in neural computation. Synapses presenting short-term depression participate in low-pass filtering, adaptation and gain control^1–4^. Tsodyks and Markram showed that cortical excitatory synapses displaying short-term depression have a limiting frequency above which changes in presynaptic firing only marginally affect the postsynaptic membrane potential^5^. Kinetics of synaptic transmission affect information transmission by tuning temporal summation^6^. This tuning is more prominent in inhibitory synapses which are characterized by slower kinetics than excitatory synapses. While short-term depression and temporal summation have numerous roles in information encoding, the contribution of these processes to computation in spontaneously active networks is less clear. A study investigating the depressing synapses at the Calyx of Held indicated that baseline firing rate considerably affects information transmitted as presynaptic activity increases^7^. Thus, information transfer via depressing synapses is dependent on short-term presynaptic dynamics, presynaptic baseline activity, and postsynaptic integration.

The basal ganglia are a group of subcortical nuclei involved in motor, limbic, and cognitive functions^8^. The entopeduncular nucleus (EP) is one of the basal ganglia output nuclei integrating synaptic information from several functional pathways within the basal ganglia. During movement, cortical information flows through the basal ganglia via the direct, indirect, and hyper-direct pathways, and converges on the EP. In the indirect pathway of the basal ganglia, globus pallidus (GP) neurons form inhibitory depressing synapses in the EP. The neurons in the GP are GABAergic and can be classified into arkypallidal neurons projecting to the striatum, and prototypical neurons projecting to the subthalamic nucleus (STN), and to the basal ganglia output nuclei, the EP and substantia nigra pars reticulata (SNr)^9,10^. In the STN, SNr, GP, and EP, GABAergic synapses from the GP exhibit short-term depression^11–15^. The steady-state depression of these synapses increases with the firing rate of the GP neuron^14^. In a previous study, we obtained paired recordings from GP neurons and showed that despite substantial depression, the constitutively active GP-GP inhibitory synapse transmitted information to postsynaptic targets as a result of temporal summation^16^. Thus, when investigating information transmission via inhibitory synapses in the basal ganglia, both plasticity and kinetics of synaptic transmission should be considered.

Classically, we view the GP as a station in the indirect pathway that serves as a source of disinhibition to allow movement suppression. However, this view is challenged by recent findings suggesting that the GP is involved in higher functions such as learning^17,18^. Although the role of the GP in motor and non-motor functions is understood better, key questions remain unanswered. The first is the fact that prototypical neurons in the GP are spontaneously active and fire over a wide range of frequencies up to 100 Hz^10,19^. These neurons change their firing rate during spontaneous movements: approximately half increase their firing rate, and half decrease their firing rate^19^. Why do GP neurons fire at such a wide range? How is information transmission during movement affected by the baseline firing rate of GP neurons? Here, we used numerical modeling and whole-cell patch-clamp recordings to shed light on the contribution of diverse presynaptic activity on information transmission in the basal ganglia indirect pathway in particular, and via GABAergic synapses in general.

## Materials and Methods

### Animals

All procedures were approved and supervised by the Institutional Animal Care and Use Committee and were following the National Institutes of Health Guide for the Care and Use of Laboratory Animals and the Bar-Ilan University Guidelines for the Use and Care of Laboratory Animals in Research. This study was approved by the Israel National Committee for Experiments in Laboratory Animals at the Ministry of Health.

### *In vitro* slice preparation

Brain slices were obtained from 15–21 day old Wistar rats as previously described^14,20–22^. Rats were killed by rapid decapitation following the guidelines of the Bar-Ilan University animal welfare committee. The brain was quickly removed and placed in ice-cold artificial cerebrospinal fluid (ACSF) containing (in mM): 125 NaCl, 2.5KCl, 15 NaHCO_3_, 1.25 Na_2_HPO_4_, 2 CaCl_2_, 1 MgCl_2_, 25 glucose, and 0.5 Na-ascorbate (pH 7.4 with 95% O_2_/5% CO_2_). In all experiments, the ACSF solution contained APV (50 μM) and CNQX (15 μM) to block NMDA and AMPA receptors, respectively (Sigma Aldrich, Cat#A6553 and Cat#C239). Thick sagittal slices (300 μm) were cut at an angle of 17 degrees to the midline on an HM 650 V Slicer (MICROM International GmbH, Germany) and transferred to a submersion-type chamber where they were maintained for the remainder of the day in ACSF at room temperature. Experiments were carried out at 37 °C while constantly perfusing the recording chamber with oxygenated ACSF.

### *In vitro* electrophysiology

*In vitro* recordings form EP neurons were done as previously described^14,22^. Individual EP neurons were visualized using infrared differential interference contrast microscopy. We performed electrophysiological recordings in the whole-cell configuration of the patch-clamp technique. Somatic whole-cell recordings were carried out using patch pipettes (4–8 MΩ) pulled from thick-walled borosilicate glass capillaries (2.0 mm outer diameter, 0.5 mm wall thickness, Hilgenberg, Mansfeld, Germany). The standard pipette solution contained (in mM): 140 K-gluconate, 10 NaCl, 10 HEPES, 4 MgATP, 0.05 SPERMIN, 5 l-glutathione, 0.2 EGTA, and 0.4 GTP (Sigma Aldrich, pH 7.2 with KOH). In some experiments, the pipette solution was supplemented with 0.2% biocytin (Sigma Aldrich, Cat#B4261) to allow staining of cellular morphology after the experiment. Under these conditions, the Nernst equilibrium potential for chloride was calculated to be −69.2 mV. The reference electrode was an Ag–AgCl pellet placed in the bath. The voltage clamp technique was used to characterize the kinetics of evoked IPSCs. The current clamp technique we used to determine the limiting frequency of the GP-EP synapse. EP neurons are spontaneously active. Thus, to clearly see and analyze postsynaptic inputs in current clamp experiments, EP neurons were hyperpolarized to the point where spontaneous firing stopped. Voltage signals were amplified by an Axopatch-200B amplifier (Axon Instruments), filtered at 5 kHz and sampled at 20 kHz. Current signals were filtered at 2 kHz and sampled at 20 kHz. We did not correct the 10 mV liquid junction potential measured under these ionic conditions. In voltage-clamp experiments, we coated the pipettes with Sylgard (Dow Corning).

Biocytin labeling was done as previously described^23^. Briefly, at the end of experiments containing biocytin in the pipette, slices were fixed overnight in cold 100 mM PBS (pH 7.4) containing 4% paraformaldehyde and washed five times in 0.1% Triton X-100 in PBS for a total of 25 min. We blocked nonspecific binding with 20% normal goat serum and 0.1% Triton X-100 in PBS for 60 min. We then incubated the slices for 24 hours at 4 °C with streptavidin-CY3 (1:1000, Sigma Aldrich, Cat#S6402) diluted in 2% normal goat serum and 0.1% Triton X-100 in PBS. Following incubation, slices were washed five times in 0.1% Triton X-100 in PBS for a total of 25 min. To label nuclei, the slices were incubated with Hoechst 33342 (1:1000, Invitrogen, Cat#H5370) for 10 min and then washed three times in 0.1% Triton X-100 in PBS for a total of 15 min. The slices were then placed on glass slides and dried for 15 minutes before being immersed in a mounting solution (Aqua Poly/Mount, Polyscience Inc., Pennsylvania, USA) and covered with a coverslip. We obtained confocal images with a Leica SP8 confocal microscope (63 × /1.4 N.A. oil objective).

We applied electrical stimulation via a monopolar 2–3 KΩ Narylene-coated stainless-steel microelectrode positioned in the GP. Stimulation pulses were biphasic 50–500 μA currents (200 μs cathodal followed by 200 μs anodal phase) using an Ag–AgCl pellet placed in the bath. Electrical stimulation of the GP may activate striatal axons going through the GP to the EP. We have previously shown that the striatal and GP synapses show differential short-term dynamics and that it is possible to distinguish them^14^. Thus, to investigate only GP evoked IPSPs, in the beginning of each recording a stimulus train of 10 pulses was delivered to the GP and the short-term dynamics of the input were characterized. Only IPSPs showing clean short-term depression were chosen for the rest of the experiment. We identified the limiting frequency of GP-EP synapses using stimulus trains of 50 pulses delivered to the GP at 1–80 Hz. To analyze changes in membrane potential induced by shifts in the activation frequency, the GP was stimulated with 50 pulses at the conditioning frequency, followed by 10 pulses at the tested frequency, followed by 50 pulses at the conditioning frequency.

### Experimental design and statistical analyses

All off-line analyses were carried out using Matlab R2016b (Mathworks) and IgorPro 6.0 (WaveMetrics, RRID: SCR_000325) on a personal computer. We obtained data for each experimental condition from at least five rats. All experimental results were pooled and displayed as the mean ± SEM unless stated otherwise. The peak amplitude of each synaptic response was calculated after subtraction of the membrane potential (in current-clamp experiments) or leak current (in voltage-clamp experiments) preceding the stimulation.

### Simulating short-term synaptic plasticity in ionotropic synapses

We based the simulations of short-term plasticity on the work of Varela *et al*.^24^, as was done previously^14,22^. This model predicts physiological results while ignoring the biological mechanisms underlying short-term plasticity. The model assumes that the postsynaptic amplitude, *A*, relies on two factors: the initial amplitude *A*_*0*_, and the depression variable *D (D* was initially set to 1).

1 $$A={A}_{0}D$$

The depression variable *D* was multiplied for each stimulus by a constant *d* representing the depression following a single action potential:

2 $$D\to Dd$$

Since *d* ≤ 1, *D* decreased with each action potential. In the model introduced by Varela^24^, after each stimulus, *D* recovered exponentially to 1 using first order kinetics with a time constant ${\tau }_{D}$:

3 $${\tau }_{D}\frac{dD}{dt}=1-D$$

Our previous findings on the GP-EP synapse suggested two time constants for recovery from depression. Thus, the GP-EP synapse was modeled to display short-term depression (*A = A*_*o*_ * d) with $d=0.5,{\tau }_{D1}=2000\,ms$, ${\tau }_{D2}=50\,ms$. In simulations of non-plastic synapses, the depression parameter d was set to 1.

### Cellular and synaptic properties

EP neurons were modeled as:

4 $$\frac{dV}{dt}=-\,\frac{1}{\tau }(V(t)-{V}_{l})+{I}_{syn}$$

5 $${I}_{syn}={g}_{GABA}(V(t)-{E}_{GABA})$$

where V is the neuron’s membrane potential, $\tau$ is the membrane time constant, and I_syn_ is the synaptic current induced by the activity GP neurons. The GABA reversal potential (E_GABA_) was set to −70 mV. The maximal GABA conductance (${g}_{GABA}$) was set to 10 nS. No voltage-gated channels were implemented in this simulation. Thus, no action potential firing was simulated and all non-linear properties of the membrane of the EP neurons were ignored.

IPSCs were modeled using an alpha function:

6 $$I(t)=\frac{t}{{\tau }_{dec}}{e}^{(1-\frac{t}{{\tau }_{dec}})}$$

where ${\tau }_{dec}$ is the time constant of decay of each evoked IPSC, and was set to 10 ms.

### Simulating dopaminergic neuromodulation

Our previous findings showed that dopamine induces a decrease in GP-EP amplitude and a decrease in short-term depression^22^. Thus, to simulate an increase in dopamine levels, *g*_*GABA*_ was decreased to 5, and d was increased to 0.7. To simulate a decrease in dopamine levels, *g*_*GABA*_ was set to 15, and d was set to 0.3. These modifications to the model parameters were based on our previous experimental results^22^.

## Results

**Figure 1:**
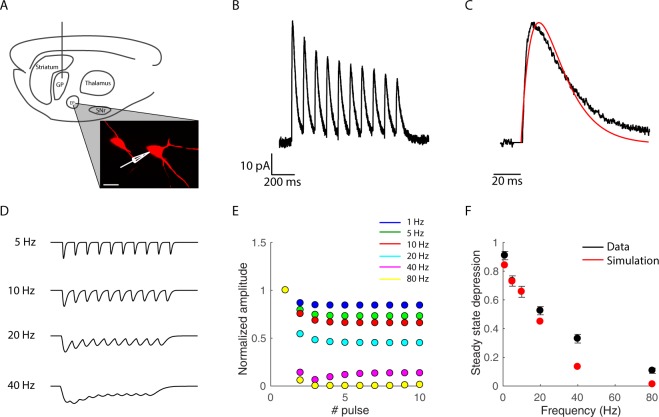
Simulation of GP-EP synapses. (**A**) Experimental design. Illustration of a sagittal brain slice containing the GP and EP. A stimulation electrode was positioned in the GP and whole cell recordings were obtained from EP neurons. A confocal image shows two biocytin filled EP neurons (scale bar = 25 μm). (**B**) Whole-cell voltage-clamp recording displaying the response of an EP neuron to stimulation of the GP at 10 Hz. (**C**) Enlargement of the first evoked IPSC in B. Red curve shows fit of an alpha function (Eq. 6) with a ${\tau }_{dec}$ of 12 ms. (**D**) simulated traces of GP evoked IPSPs induced by activation of the synapse at 5, 10, 20 and 40 Hz. (**E**) normalized IPSP amplitude evoked by each activation of the simulated GP synapse at different frequencies. (**F**) steady-state depression as a function of the time interval between pulses. Black traces represent population average of the IPSP amplitude evoked by each pulse recorded during GP stimulation at different frequencies. Means ± SEM. Red traces represent data from the simulation.

We investigated how short-term dynamics and IPSC kinetics contributed to information transmission in constitutively active GABAergic synapses. Our model system of choice was the synapse between the GP and EP in the indirect pathway of the basal ganglia. We performed voltage-clamp recordings from EP neurons and recorded their response to repetitive stimulation of the GP (Fig. 1A,B). As expected, GP-evoked IPSCs displayed short-term depression. We revealed that GP evoked IPSCs were characterized by slow decay kinetics by fitting the evoked currents to an alpha function (Eq. 6) (${\tau }_{dec}=9.8\pm 0.2\,ms$, n = 20, Fig. 1C). The GP-EP synapse displays short-term depression at all frequencies, and steady-state depression increases exponentially with the presynaptic firing rate^11,14^. We used these parameters to construct a data-driven model of the GP-EP synapse (Fig. 1D–F). Figure 1D displays numerically simulated IPSPs evoked by different presynaptic frequencies. The steady-state depression of the GP-EP synapse in our model increased exponentially with the presynaptic firing rate, in a similar manner to that found in slices^11,14^ (Fig. 1E,F).

### Limiting frequency of GP-EP synapses

**Figure 2:**
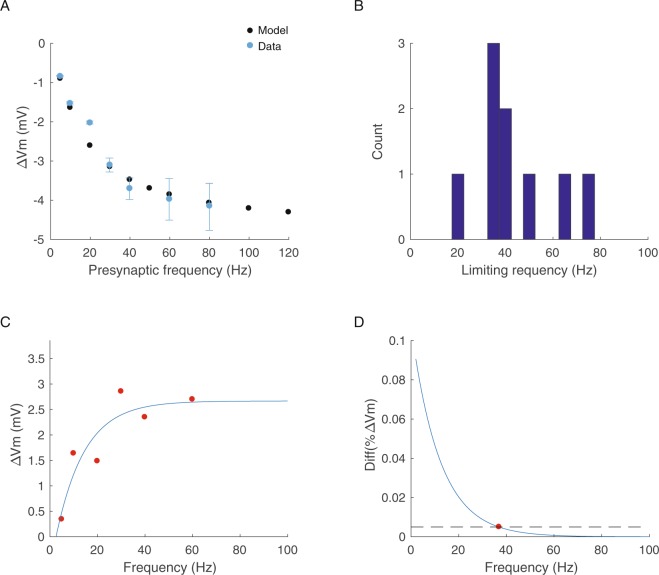
Steady state inhibition induced by GP activity. (**A**) Average change in EP membrane potential during steady state inhibition induced by simulated regular stimulation (black) and experimental data (blue). The GP was stimulated at 5 Hz (n = 24), 10 Hz (n = 24), 20 Hz (n = 24), 30 Hz (n = 11), 40 Hz (n = 9), 60 Hz (n = 5) and 80 Hz (n = 6). (**B**) Distribution of the limiting frequency calculated for 9 individual GP-EP synapses, calculated from GP evoked IPSPs. (**C**,**D**) Example of calculation of the limiting frequency of a GP-EP synapse. (**C**) Change of the EP membrane potential as a function of the stimulation frequency of the GP (red dots). The data were fitted with an exponential function (blue trace). (**D**) The limiting frequency was extracted as the activation frequency that induced a change in Vm that was smaller than 5%.

We used this model to investigate how a GP synapse could change the membrane potential of an EP neuron. Previous studies have shown that a limiting frequency, above which increases in the presynaptic firing rate do not affect the membrane potential of the postsynaptic neuron due to the increase in steady-state depression, is characteristic of depressing synapses^5^. The limiting frequency of cortical synapses was found to be ~15–25 Hz^5^. Interestingly, the limiting frequency of depressing synapses in the chick auditory system was found to be ~250 Hz^25^, suggesting that different depressing synapses may act over different frequency ranges. Thus, we used our model to determine the limiting frequency of GP-EP synapses. We activated the simulated synapse at various frequencies with 50 pulses, which were enough for the IPSP amplitude to reach a steady state. We measured the change in the simulated membrane potential during the steady-state and found that the limiting frequency of the simulated synapse was ~45 Hz (Fig. 2A). To validate our findings, we obtained current-clamp recordings from EP neurons and recorded the change in membrane potential induced by electrical stimulation of the GP at different frequencies (Fig. 2A). We stimulated the GP with trains of 50 pulses at 5–80 Hz and recorded the response of EP neurons in current-clamp mode (n = 28). Our electrophysiological recordings matched the simulated data, showing similar changes in the EP membrane potential (Fig. 2A). We calculated the limiting frequency of individual synapses and found that it was 44 ± 5 Hz (Fig. 2B–D, n = 9). Thus, our recordings corroborated our conclusion that the limiting frequency of GP-EP synapses for information transmission is 45 Hz.

### Baseline firing rate affects the level and variance of GP induced inhibition

**Figure 3:**
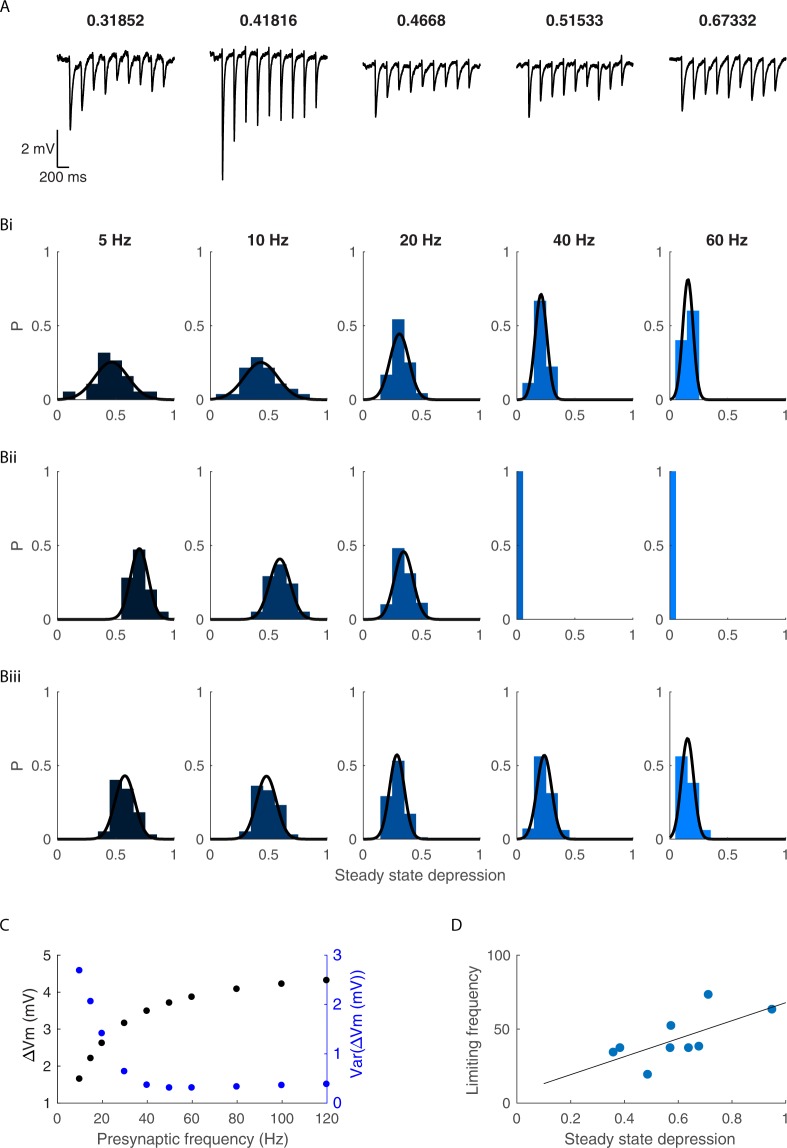
Variability of steady state depression changes as a function of activation frequency. (**A**) Example traces from EP neurons during stimulation of the GP at 5 Hz. The steady state depression is indicated above each recording. (**B**) The distribution of the steady state depression for each of the stimulation frequencies extracted from the experimental data (i), from simulated IPSPs evoked by regular stimulation (ii) and from simulated IPSPs evoked by Poisson stimulation (iii). (**C**) The mean and variance of the change in EP membrane potential as a function of the activation frequency. (**D**) The limiting frequency of GP-EP synapses as a function of the steady state depression (measured from stimulation of the GP at 10 Hz). R^2^ = 0.44, p = 0.049.

Our whole-cell recordings revealed that different GP-EP synapses have different limiting frequencies, with a distribution centered around 45 Hz (Fig. 2B). We examined our data and measured the variance of steady-state depression (SSD) for each activation frequency (Fig. 3A,B). Our data showed that the variance of SSD decreases as the activation frequency increases. This reduced variance results from the two main factors affecting SSD: the presynaptic level of depression, which is a function of the depression time constant and the depression factor D, and the postsynaptic temporal filtering, which is a function of the membrane time constant. For a single EP neuron, the properties of GP synapses differ, whereas the membrane time constant does not. Thus, at low frequencies, the effect of temporal summation is small, and the SSD is mostly a function of presynaptic factors. However, as the presynaptic firing increases, the effect of temporal summation on depression increases, and the resulting SSD does not vary. When we added variance to the model, we found that similar to our experimental results, the variance decreased as the activation frequency increased (Fig. 3B,C). As expected, the limiting frequency of the GP-EP synapses changed as a function of the steady-state depression (Fig. 3D). These findings suggest that the baseline firing rate of a GP neuron affects not only the membrane potential of EP neurons but also the variability of the membrane potential across the EP population.

### Gain control in the indirect basal ganglia pathway

GP neurons are spontaneously active and show brief changes in firing rate during movement^19,26^. Thus, a more realistic activity pattern would be one that shifts between frequencies. Previous studies have shown that gain control in depressing synapses results with no transmission of the net change in firing rate to the postsynaptic neuron^27^. We used our model to investigate how the baseline activity of a GP neuron could influence information transmission to the EP. We activated the simulated GP synapse at different frequencies, induced a transient change in the activation frequency, and measured the induced change in inhibition (Fig. S1A). We quantified the change in inhibition during the transient change by measuring the mean change in postsynaptic membrane potential. Our simulations showed that the mean inhibition during the transient frequency change was nearly independent of the baseline frequency, as opposed to a simulation in which the synapse had no short-term depression (Fig. S1B,C). This result is in line with a model of gain control. We observed small changes when the baseline frequencies were lower than 45 Hz, the limiting frequency of these synapses (Fig. S1Bi,Ci). Thus, acting in isolation, the GP-EP synapse is consistent with a model of gain control where the baseline firing rate of a GP neuron affects postsynaptic inhibition at firing rates that are below the limiting frequency.

**Figure 4:**
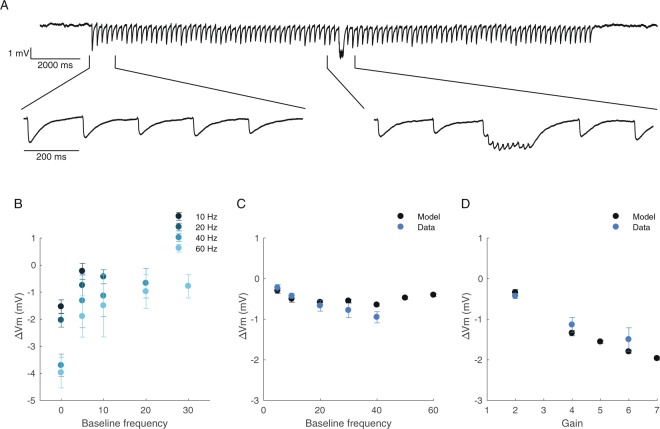
Whole-cell recordings of the response of EP neurons to transient increases in activation frequency of the GP. (**A**) Example trace showing the membrane potential of an EP neuron during stimulation of the GP. The GP was stimulated with 50 pulses at 5 Hz, followed by 10 pulses at 60 Hz, followed by 50 pulses at 5 Hz. (**B**) Average change in membrane potential as a function of the baseline frequency. The change in membrane potential (ΔVm) was measured during the 2^nd^ activation frequency. (**C**) Average change in membrane potential induced by multiplication of the activation frequency by 2 as a function of the baseline frequency. (**D**) Average change in membrane potential induced by multiplication of the activation frequency by different gains, baseline frequency of 10 Hz.

We carried out whole-cell recordings from EP neurons during electrical stimulation of the GP. The GP was stimulated with 50 pulses at a baseline frequency, followed by 10 pulses at a second activation frequency, and then with 50 pulses at the original baseline frequency (Fig. 4). Figure 4A shows the change in the membrane potential of an EP neuron during increased activation of the GP. We measured the steady-state inhibition during the second frequency and found a minimal dependence of the induced inhibition on the baseline frequency, in line with model predictions (Fig. 4B–D). Thus, these electrophysiological recordings confirm that GP-EP synapses mediate gain control in the indirect pathway.

### Integration of multiple inputs

**Figure 5:**
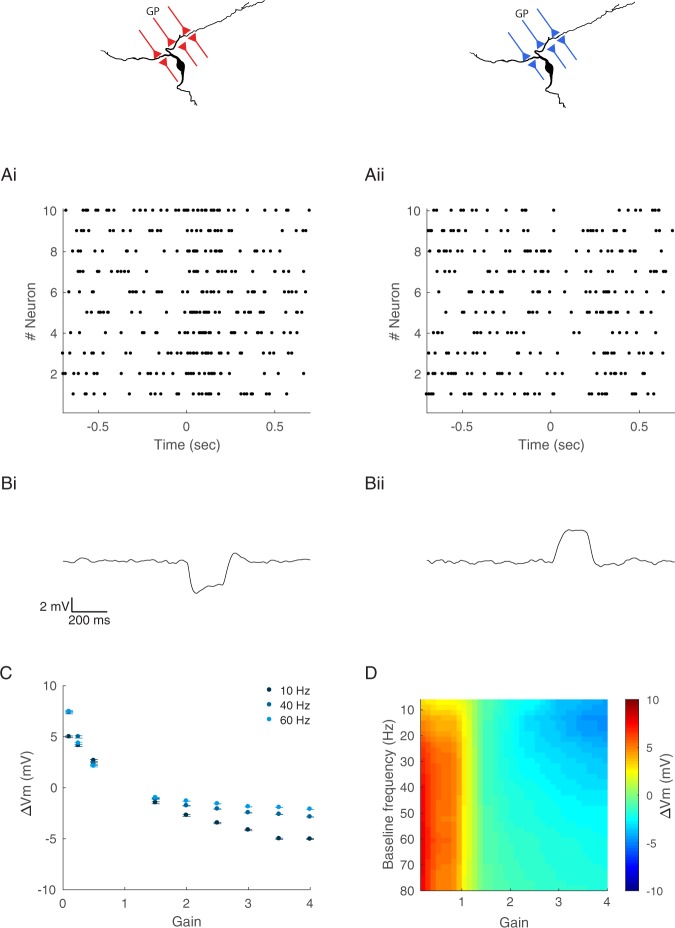
Integration of multiple inputs is mediated by gain control of the GP-EP synapse. GP inputs are labeled red or blue for neurons increasing or decreasing their firing rate, respectively. (**A**) Raster plot of the activity of 10 simulated GP neurons that increase (i) or decrease (ii) their firing rate for 200 ms. In these examples the baseline firing rate of all neurons was 20 Hz and the gain was set to 3 (i) or 1/3 (ii). (**B**) Predicted membrane potential of an EP neuron that receives input from 10 GP neurons in (**A**). (**C**) Predicted change in membrane potential as a function of the multiplication factor, calculated for baseline frequencies of 10, 40 and 60 Hz. (**D**) Predicted change in membrane potential as a function of the multiplication factor and the baseline firing rate.

Our findings thus far show that the GP-EP synapse appears to mediate gain control. However, there is enormous convergence in the rat indirect pathway, in which 46,000 neurons of the GP converge onto 3200 neurons of the EP, suggesting that each neuron in the EP integrates inputs from multiple GP neurons^28^. In the EP, integration of multiple inputs is expected to be sublinear, since the reversal potential of GABA, which is ~−75 mV in the EP^14^, is not too far from the postsynaptic membrane potential. Thus, we used our model to predict how EP neurons would integrate information from multiple GP neurons. Each EP neuron in the model received input from 10 GP neurons that either increased or decreased their firing rate for 200 ms (Fig. 5A,B). The EP neurons were also injected with a constant current to ensure a uniform baseline membrane potential of ~−50 mV (Fig. 5). We then measured the induced change in postsynaptic inhibition during the second activation frequency (Fig. 5C,D). Our simulations demonstrated that during the integration of multiple inputs, the induced change in the postsynaptic membrane potential was not only more significant but differentially dependent on the baseline firing rate of the presynaptic neurons (Fig. 5B–D). Furthermore, when an EP neuron integrated multiple inputs, the induced change in inhibition depended on the baseline frequency for both high and low gains (Fig. 5C,D). Figure 5D depicts the predicted change in membrane potential as a function of both the gain and the baseline frequency.

The effect of increased and decreased GP activity on the postsynaptic membrane potential differed in their dynamics. Decreasing GP firing resulted in the excitation of the postsynaptic neurons that lasted for the entire duration of the change in GP firing. The evoked excitation was constant and did not change significantly during that period (Fig. 5Bii). However, increasing the GP firing resulted in a brief inhibition that decayed with time (Fig. 5Bi). This result was expected since GP synapses show short-term depression; hence, their inhibitory effect decreases with their activation.

### Integration of multiple heterogeneous inputs leads to several modes of EP modulation

**Figure 6:**
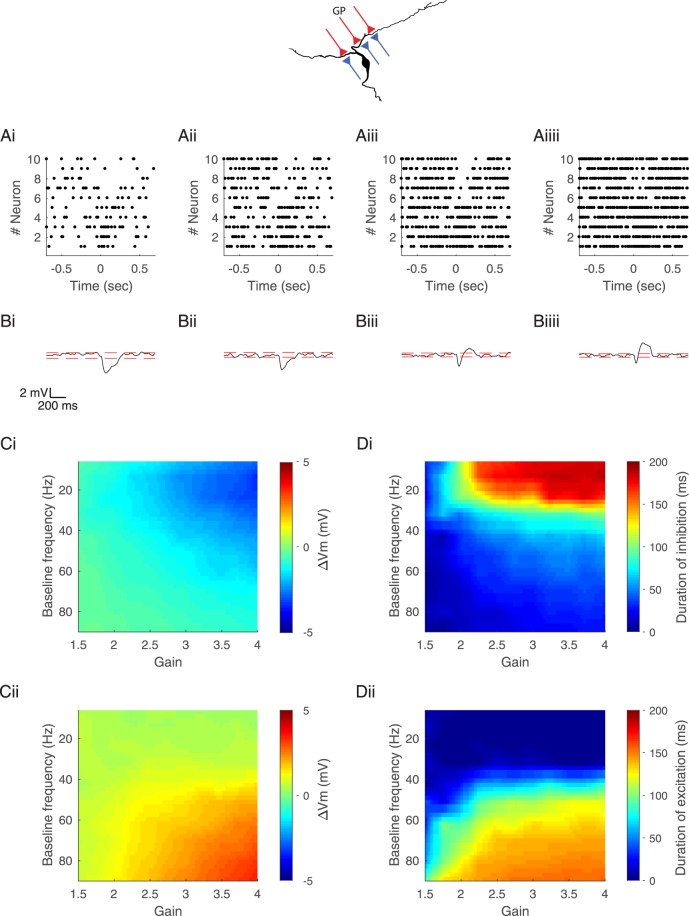
Integration of heterogeneous inputs. GP inputs are labeled red or blue for neurons increasing or decreasing their firing rate, respectively. (**A**) Raster plot of the activity of 10 simulated GP neurons that changed their firing rate for 200 ms. Half of the neurons increased their firing rate and half of the neurons decreased their firing rate. The four examples differ in terms of their baseline firing rate: 10 (i), 20 (ii), 40 (iii) and 60 Hz (iiii). (**B**) Predicted membrane potential of an EP neuron that receives input from 10 GP neurons in (**A**). Dashed red line indicates two SD below the mean membrane potential. (**C**) Predicted change in membrane potential as a function of the multiplication factor and the baseline firing rate. (**D**) Duration of the inhibitory phase (i) and excitatory phase (ii) induced by the change in presynaptic activity. The duration of inhibition was defined as the time during which the postsynaptic membrane potential was significantly hyperpolarized (more negative than 2 SD below the mean membrane potential).

Our findings suggest that GP-EP modulation depends on the number of GP neurons changing their activity and predict different dynamics for EP neurons integrating multiple inputs from GP neurons, which either increase or decrease their firing rate. However, EP neurons receive heterogeneous input from the GP and are innervated by GP neurons that increase their firing rate, as well as GP neurons that decrease their firing rate. Thus, we investigated how EP neurons integrate multiple opposing inputs. To do so, we simulated the change in membrane potential of an EP neuron that received input from 5 GP neurons that increased their firing rate and 5 GP neurons that decreased their firing rate for an equivalent magnitude (Fig. 6A). Our simulations revealed that these opposing inputs did not altogether cancel out. Instead, the postsynaptic membrane potential showed a dynamic change depending on the baseline firing rate in the GP (Fig. 6B).

Integration of heterogeneous inputs resulted in prolonged inhibitory phases when the baseline frequencies were low and prolonged excitatory phases when the baseline frequencies were high (Fig. 6C,D). For medium baseline frequencies, integration of heterogeneous inputs resulted in a brief inhibitory phase followed by a brief excitatory phase. The duration and magnitude of the inhibitory phase decreased as the baseline firing rate increased (Fig. 6C,D). Conversely, the duration and magnitude of the excitatory phase increased as the baseline firing rate increased. Thus, the combination of several broad changes in GP activity can lead to one of several very different outcomes in the EP. Our simulations predicted an unambiguous mapping from the presynaptic baseline frequency to the type of postsynaptic response. Whereas low and high baseline frequencies resulted in prolonged inhibition or excitation, medium baseline frequencies resulted in temporally focused hyperpolarization followed by depolarization in the membrane potential of an EP neuron. Additionally, the magnitude and temporal component of the GP evoked inhibitory and excitatory phases depended heavily on the baseline firing rate of the presynaptic neurons.

### Dopamine induces a shift in the effects of the GP baseline firing rates

**Figure 7:**
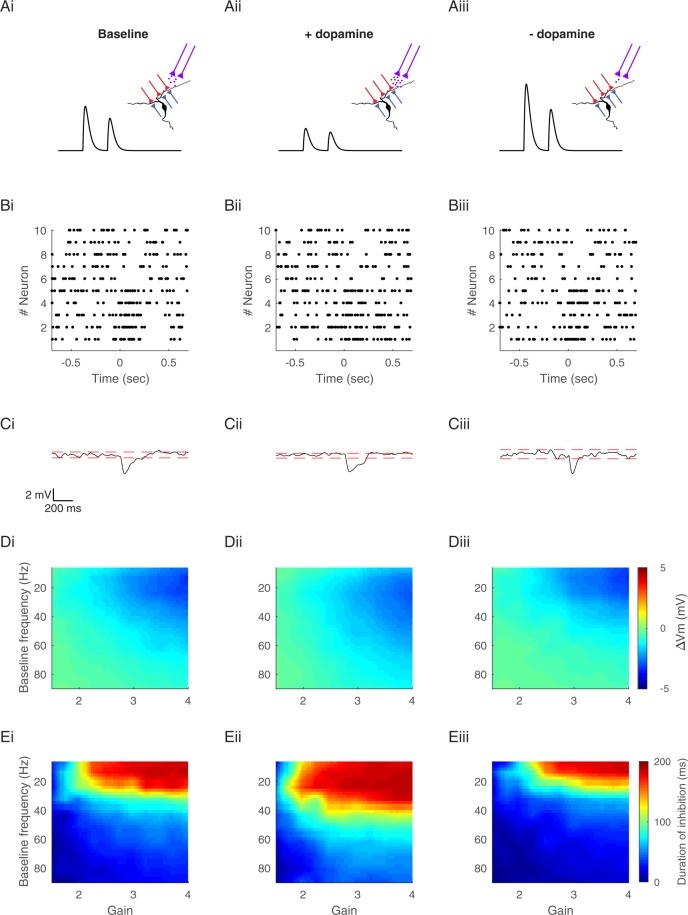
Dopaminergic modulation of integration of pallidal inputs. (**A**) Illustration of dopaminergic neuromodulation. GP inputs are labeled red or blue for neurons increasing or decreasing their firing rate. Dopaminergic input from the SNc is labeled in magenta. Black traces illustrate the effect of dopamine on GABAergic conductance of GP-EP synapses. (i), basal dopamine levels, parameters are consistent with the simulations so far. (ii), increased dopamine concentration, simulated by a decrease in the amplitude and depression of GP-EP synapses. (iii), decreased dopamine concentration, simulated by an increase in amplitude and depression of GP-EP synapses. (**B**) Raster plot of the activity of 10 simulated GP neurons that change their firing rate for 200 ms. Half of the neurons increased their firing rate and half of the neurons decreased their firing rate. The baseline firing rate of all neurons was 20 Hz. (**C**) Predicted membrane potential of an EP neuron that receives input from 10 GP neurons in B. Dashed red line indicates two SD from the mean membrane potential. (**D**) Predicted change in membrane potential as a function of the multiplication factor and the baseline firing rate. (**E**) Duration of the inhibitory phase induced by the change in presynaptic activity. The duration was defined as the time during which the postsynaptic membrane potential was significantly hyperpolarized (more negative than 2 SD below the mean membrane potential).

Our findings thus far show that the broad range of baseline firing rates in the GP enables different modes of modulation of neural activity in the EP. These modes of action result from the short-term depression and temporal summation of GP-EP synapses. The shift between the different modes occurs at the limiting frequency of GP-EP synapses. However, the limiting frequency, a function of steady-state depression, may change due to neuromodulation. We recently showed that dopamine, a key neuromodulator of basal ganglia function, affects synaptic transmission in the GP-EP synapse by decreasing the amplitude of synaptic transmission and reducing short-term depression^22^. Thus, we simulated the effects of changes in dopamine concentration on information transmission from the GP to the EP (Fig. 7A,B). Our simulations predicted that changes in dopamine levels would induce a shift in the baseline frequencies that underlie the different modes of action on the EP. An increase in dopamine concentration, simulated by decreased amplitude and reduced depression, should increase the range of baseline frequencies that underlie prolonged inhibition. Conversely, a decrease in dopamine concentration, simulated by an increased amplitude and increased depression, should decrease the range of baseline frequencies that underlie prolonged inhibition (Fig. 7C,D). Thus, our simulations predicted that the levels of dopamine would affect the response in the EP and its dependence on GP baseline firing rates.

### Different onsets of changes in GP activity

GP neurons that decrease their firing rate and the GP neurons that increase their firing rate do so at significantly different time scales during spontaneous movements^19^. Specifically, neurons increasing their firing rate initiate earlier than those decreasing their firing rate. We thus incorporated a delay of 50 ms into our model (Fig. S2A,B). Our simulations predicted that adding a delay would increase the magnitude and duration of both the inhibitory and excitatory phases (Fig. S2C,D). Furthermore, incorporating a delay was expected to expand the range of frequencies displaying both phases. For example, with no delay, integration of multiple inputs with a baseline firing rate of 20 Hz can induce a brief inhibition in the EP. However, incorporating a delay of 50 ms induces the emergence of a brief excitatory phase following the inhibitory phase (Fig. S2). Thus, incorporating a delay increases the frequency band that induces an inhibition-excitation response in the EP, and reduces the frequency bands which induce prolonged excitation or inhibition.

## Discussion

**Figure 8:**
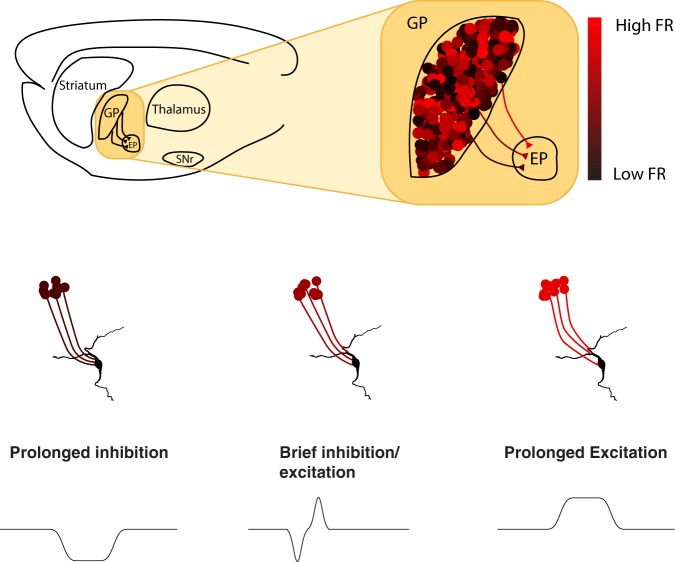
Short-term depression and temporal summation allow different baseline frequencies to underlie different modes of action in the globus pallidus. Upper panel: schematic illustration of the basal ganglia indirect pathway in the rat brain. Neurons in the GP project to the EP and fire at a wide range of frequencies. Lower panel: different modes of action are enabled by the different baseline frequencies. Low baseline firing rate in the GP allows GP neurons to induce prolonged inhibition in the EP, medium baseline frequencies allow GP neurons to induce brief inhibition or excitation in the EP, high baseline frequencies allow GP neurons to induce prolonged excitation in the EP.

We explored the role of short-term depression on information transmission in a constitutively active GABAergic synapse of the basal ganglia’s indirect pathway. We demonstrated that the broad frequency range of GP neurons leads to different levels of inhibition as well as variance in the postsynaptic membrane potential. The average limiting frequency of GP-EP synapses suggests that the inhibitory control of GP over neurons in the EP depends on the baseline firing rate of the GP at frequencies below 45 Hz. During the integration of multiple heterogeneous inputs, different baseline frequencies of GP neurons underpinned different modes of EP modulation. The direction, amplitude, and duration of the change in postsynaptic membrane potential depended on the baseline firing rate in the GP. Thus, due to short-term depression and slow kinetics of GP-EP synapses, the extensive range of GP firing rates enabled different modes of information transmission, in which the magnitude and temporal features of postsynaptic modulation changed as a function of present and past firing rates of the neurons in the GP (Fig. 8).

The classical view of basal ganglia function suggests that the two main pathways of this system may have opposing effects on movement: information flow in the direct pathway facilitates movement, whereas information flow in the indirect pathway inhibits movement. Many findings challenged this model, suggesting that the roles of these two pathways may not be as segregated. Accumulating evidence shows that a similar amount of direct and indirect striatal spiny projection neurons correspondingly change their activity during movement^29,30^. The GP, classically viewed as a station in the indirect pathway, also receives input from striatal spiny projection neurons of the direct pathway^31,32^. Moreover, during movement, half of the GP neurons increase their activity and thus do not adhere to their classical role of disinhibition^19^. Furthermore, *in vivo* studies in rodents and primates have shown the involvement of the GP in non-motor functions, such as learning^17,18^. In summary, the indirect pathway appears to encode multiple types of information and does not solely serve as a source of disinhibition.

In the indirect pathway of the basal ganglia, GP neurons form GABAergic depressing synapses in the EP. EP neurons are spontaneously active and fire *in vivo* at ~26 Hz^33,34^. We have previously shown that stimulation of the GP at 40 or 80 Hz can silence spontaneously active EP neurons and that stimulation of the GP at lower frequencies can induce a transient decrease in firing rate and spike time entropy of the postsynaptic neuron^14,22^. Others have shown that single stimulation of the GP in brain slices can synchronize EP neurons^11^. However, these studies did not consider that GP neurons are spontaneously active, and that GP synapses are consequently substantially depressed. Thus, the ability of GP neurons to synchronize or silence the activity of neurons in the EP might be dependent on the baseline activity of the presynaptic neuron. Moreover, these studies did not consider the convergence in this pathway, which is evident from the massive decrease in the number of neurons, as well as from anatomical studies suggesting that each EP neuron receives input from multiple GP neurons^28,35–37^.

GP neurons fire over an extensive frequency range, but the functionality of this range is not evident^10,19^. We have previously shown that, in primates, injection of bicuculline to the GPe leads to an average increase in GPe firing rate from 84 Hz to 113 Hz^38^. However, in the same animals, the average firing rate of GPi neurons did not change, questioning the ability of GPe neurons to inhibit neurons in the GPi^38^. Thus, the first goal of the current study was to investigate the potential role of the different ongoing firing frequencies in the GP. Our simulations and whole-cell recordings showed that the limiting frequency of GP-EP synapses is ~45 Hz, which suggests that pallidal input can introduce different levels of inhibition to the EP when firing at frequencies below 45 Hz. We further found a pronounced variance in steady-state depression, which decreased with the activation frequency of the GP. Thus, the variability between neurons in the EP is likely to decrease as the activity in the GP increases.

During movement, GP neurons briefly change their firing rate. In other systems, synapses displaying short-term depression mediate gain control, such that the change in postsynaptic membrane potential does not depend on the baseline firing rate of the presynaptic neuron^27^. In line with gain control model, our simulations and whole-cell recordings show that when a GP neuron changes its firing rate, the induced inhibition manifests very little dependence on the baseline firing rate. However, when several neurons change their activity, the input-output relationship changes. We demonstrated that the integration of opposing inputs with equivalent magnitude did not result in zero change but instead led to dynamic change in the postsynaptic membrane potential. The direction, duration, and magnitude of the postsynaptic response changed as a function of the gain, and the baseline frequency of the presynaptic neuron. Specifically, during synaptic integration of heterogeneous inputs, GP neurons firing at low firing rates can induce prolonged inhibition. This postsynaptic inhibition narrows as the presynaptic frequency increases. Over a band of presynaptic baseline frequencies, an excitatory phase appears after the inhibitory phase. As the presynaptic baseline frequency increases, the excitatory phase becomes larger. When the baseline frequency in the GP is high, integration of multiple inputs in the EP induces prolonged excitation. Thus, while individual GP-EP synapses mediate gain control, when we consider the integration of multiple opposing inputs, the synaptic properties lead to a completely different outcome, which allows different baseline frequencies to transmit markedly different signals to the EP.

Dopamine, a key neuromodulator involved in learning, reward and motor control, modulates neural activity throughout the basal ganglia^39,40^. Recent findings have highlighted the importance of dopamine at the output level of the basal ganglia. All the dopamine receptors are expressed in the EP on presynaptic and postsynaptic elements^41^. *In vivo*, dopamine gates information flow in the EP and is necessary for releasing movements^42,43^. We have shown elsewhere that dopamine acts in the EP by differentially modulating synaptic transmission from the striatum and GP^22^. These findings indicated that blocking dopamine increases basal transmission and depression in the GP-EP synapse^22^. Here we suggest that changes in dopamine levels can shift the action of GP neurons from rate coding to gain modulation. Our simulations suggest that changes in dopamine levels may result in a shift in the mapping of the baseline presynaptic firing rate to the postsynaptic response. An increased level of dopamine is expected to decrease the level of synaptic depression and thus increase the range of baseline frequencies that can induce prolonged inhibition. Conversely, when the level of dopamine decreases, this frequency band also decreases^13,15^.

Here we investigated GP modulation of activity in the EP. Is this type of modulation specific to EP neurons, or does the GP affect similarly all of its targets? GP axons form synapses with similar short-term dynamics in the STN and SNr^13,15^. Moreover, GP evoked IPSCs in the STN and SNr are depressed by dopamine via D2 like receptors, similar to these inputs in the EP^15,22,44^. STN and SNr neurons are spontaneously active, and their activity might be similarly modulated by GP input. A previous study of GP-STN synapses investigated the effects of short-term depression and integration of multiple inputs on information transmission in this pathway^45^. This study showed that synchronous activation of GP inputs can silence neurons in the STN, whereas non-synchronized activity of multiple GP neurons disrupts the autonomous firing of these neurons, leading to decreased firing rate and increased CV^45^. It should be noted that while the properties of single synapses appear to be similar in the three nuclei, the connectivity pattern is very different between the STN and the output nuclei. Unlike the EP and SNr, subthalamic neurons innervate the GP, creating a feedback loop with more complicated dynamics^46,47^. Thus, future studies should investigate the limiting frequency of GP-STN and GP-SNr synapses, and the effect of different baseline firing rate on postsynaptic activity, to determine if GP neurons affect these three structures in a similar manner, or if the modulation is target dependent.

In the present study all experiments were performed on slices obtained from relatively young animals of postnatal days 15–21 (P15-P21). Several studies have shown that synaptic properties can change during development^13,48,49^. A study investigating the properties of inhibitory synapses in the medial nucleus of the trapezoid body (MNTB) showed that the amplitude and kinetics of evoked IPSCs change dramatically between P5-7 and P25^48^. Another study of excitatory synapses in the MNTB showed changes in short-term plasticity of these synapses during the same developmental period^49^. While today there is no comparative data of the GP-EP synapse at different developmental stages, it has been shown that in the SNr, GP synapses show short-term depression with stable PPR across development from P14 to P20^13^, suggesting that our findings may be relevant for older animals. However, as synaptic changes are possible at later stages of development, it would be interesting to see if the findings we have here are similar in older animals.

The EP, an output nucleus of the basal ganglia, forms inhibitory synapses in the thalamus, thus controlling thalamic ability to modulate movement^50,51^. During movement, neurons in the EP integrate inputs from all three basal ganglia pathways. In the direct pathway, EP neurons receive inhibitory input directly from the striatum such that the continuous inhibition from the EP is paused, allowing activation of the thalamus and subsequently the cortex. In the hyperdirect pathway, glutamatergic input from the STN is transmitted directly to the EP, increasing thalamic inhibition to prevent or stop the execution of movement. Assigning a simple function to information transmission in the indirect pathway is less clear since it travels through multiple stations. As striatal and subthalamic inputs converge in the GP, half of the neurons in the GP are excited and half inhibited, which raises the question of whether the activity of these neurons affects activity in the EP. Here, we suggest that differential changes in GP activity may be transmitted to the EP. We show that due to synaptic and network properties, integration of either homogeneous or heterogeneous inputs can result in several significant changes in postsynaptic excitability, which vary in direction, duration and magnitude.

## Supplementary information

supplementary information

## Data Availability

Data sets and computer code can be freely obtained from the authors by request.

## References

[1] Anwar H, Li X, Bucher D, Nadim F (2017). Functional roles of short-term synaptic plasticity with an emphasis on inhibition. Curr. Opin. Neurobiol..

[2] Chance FS, Nelson SB, Abbott LF (1998). Synaptic depression and the temporal response characteristics of V1 cells. J Neurosci.

[3] Fuhrmann G, Segev I, Markram H, Tsodyks M (2002). Coding of Temporal Information by Activity-Dependent Synapses. J. Neurophysiol..

[4] Chung S, Li X, Nelson SB (2002). Short-term depression at thalamocortical synapses contributes to rapid adaptation of cortical sensory responses *in vivo*. Neuron.

[5] Tsodyks M, Markram H (1997). The neural code between neocortical pyramidal neurons depends. Proc. Nat. Acad. Sci. USA.

[6] Banitt Y, Martin KA, Segev I (2005). Depressed responses of facilitatory synapses. J Neurophysiol.

[7] Hermann J, Pecka M, von Gersdorff H, Grothe B, Klug A (2007). Synaptic transmission at the calyx of Held under *in vivo* like activity levels. J. Neurophysiol..

[8] Mink JW (1996). The basal ganglia: Focused selection and inhibition of competing motor programs. Prog. Neurobiol..

[9] Mallet N (2012). Dichotomous organization of the external globus pallidus. Neuron.

[10] Abdi, A. *et al*. Prototypic and Arkypallidal Neurons in the Dopamine-Intact External Globus Pallidus. 35, 6667–6688 (2015). 10.1523/JNEUROSCI.4662-14.2015 PMC4412890 25926446

[11] Kita H (2001). Neostriatal and globus pallidus stimulation induced inhibitory postsynaptic potentials in entopeduncular neurons in rat brain slice preparations. Neuroscience.

[12] Sims RE, Woodhall GL, Wilson CL, Stanford IM (2008). Functional characterization of GABAergic pallidopallidal and striatopallidal synapses in the rat globus pallidus *in vitro*. Eur J Neurosci.

[13] Connelly WM, Schulz JM, Lees G, Reynolds JN (2010). Differential short-term plasticity at convergent inhibitory synapses to the substantia nigra pars reticulata. J Neurosci.

[14] Lavian H, Korngreen A (2016). Inhibitory short-term plasticity modulates neuronal activity in the rat entopeduncular nucleus *in vitro*. Eur. J. Neurosci..

[15] Baufreton J, Bevan MD (2008). D2-like dopamine receptor-mediated modulation of activity-dependent plasticity at GABAergic synapses in the subthalamic nucleus. J Physiol.

[16] Bugaysen J, Bar-Gad I, Korngreen A (2013). Continuous modulation of action potential firing by a unitary GABAergic connection in the globus pallidus *in vitro*. J Neurosci.

[17] Schechtman E, Noblejas MI, Mizrahi AD, Dauber O, Bergman H (2016). Pallidal spiking activity reflects learning dynamics and predicts performance. Proc. Natl. Acad. Sci..

[18] Gittis AH (2014). New Roles for the External Globus Pallidus in Basal Ganglia Circuits and Behavior. J. Neurosci..

[19] Dodson PD (2015). Distinct Developmental Origins Manifest in the Specialized Encoding of Movement by Adult Neurons of the External Globus Pallidus Distinct Developmental Origins Manifest in the Specialized Encoding of Movement by Adult Neurons of the External Globus Pallid. Neuron.

[20] Lavian H, Ben-Porat H, Korngreen A (2013). High and low frequency stimulation of the subthalamic nucleus induce prolonged changes in subthalamic and globus pallidus neurons. Front. Syst. Neurosci..

[21] Stuart GJ, Dodt HU, Sakmann B (1993). Patch-clamp recordings from the soma and dendrites of neurons in brain slices using infrared video microscopy. Pflugers Arch.

[22] Lavian H (2017). Dopaminergic Modulation of Synaptic Integration and Firing Patterns in the Rat Entopeduncular Nucleus. J. Neurosci..

[23] Grobman M (2018). A Mirror-Symmetric Excitatory Link Coordinates Article A Mirror-Symmetric Excitatory Link Coordinates Odor Maps across Olfactory Bulbs and Enables Odor Perceptual Unity. Neuron.

[24] Varela JA (1997). A quantitative description of short-term plasticity at excitatory synapses in layer 2/3 of rat primary visual cortex. J Neurosci.

[25] Cook DL, Schwindt PC, Grande LA, Spain WJ (2003). Synaptic depression in the localization of sound (vol 421, pg 66, 2003). Nature.

[26] DeLong MR (1971). Activity of pallidal neurons during movement. J Neurophysiol.

[27] Abbott LF, Varela JA, Sen K, Nelson SB (1997). Synaptic depression and cortical gain control. Science (80-.)..

[28] Oorschot DE (1996). Total number of neurons in the neostriatal, pallidal, subthalamic, and substantia nigral nuclei of the rat basal ganglia: a stereological study using the cavalieri and optical disector methods. J Comp Neurol.

[29] Markowitz Jeffrey E., Gillis Winthrop F., Beron Celia C., Neufeld Shay Q., Robertson Keiramarie, Bhagat Neha D., Peterson Ralph E., Peterson Emalee, Hyun Minsuk, Linderman Scott W., Sabatini Bernardo L., Datta Sandeep Robert (2018). The Striatum Organizes 3D Behavior via Moment-to-Moment Action Selection. Cell.

[30] Cui G (2013). Concurrent activation of striatal direct and indirect pathways during action initiation. Nature.

[31] Parent A, Charara A, Pinault D (1995). Single striatofugal axons arborizing in both pallidal segments and in the substantia nigra in primates. Brain Res..

[32] Kawaguchi Y, Wilson CJ, Emson PC (1990). Projection subtypes of rat neostriatal matrix cells revealed by intracellular injection of biocytin. J. Neurosci..

[33] Ruskin DN, Bergstrom DA, Walters JR (2002). Nigrostriatal lesion and dopamine agonists affect firing patterns of rodent entopeduncular nucleus neurons. J Neurophysiol.

[34] Benhamou L, Cohen D (2014). Electrophysiological characterization of entopeduncular nucleus neurons in anesthetized and freely moving rats. Front Syst Neurosci.

[35] Bevan MD, Clarke NP, Bolam JP (1997). Synaptic integration of functionally diverse pallidal information in the entopeduncular nucleus and subthalamic nucleus in the rat. J Neurosci.

[36] Kincaid AE, Penney JB, Young AB, Newman SW (1991). Evidence for a projection from the globus pallidus to the entopeduncular nucleus in the rat. Neurosci Lett.

[37] Bevan MD, Booth PA, Eaton SA, Bolam JP (1998). Selective innervation of neostriatal interneurons by a subclass of neuron in the globus pallidus of the rat. J Neurosci.

[38] Bronfeld M (2010). Bicuculline-induced chorea manifests in focal rather than globalized abnormalities in the activation of the external and internal globus pallidus. J. Neurophysiol..

[39] Beninger RJ (1983). The role of dopamine in locomotor activity and learning. Brain Res.

[40] Schultz W, Apicella P, Scarnati E, Ljungberg T (1992). Neuronal activity in monkey ventral striatum related to the expectation of reward. J Neurosci.

[41] Lavian H (2018). Dopamine receptors in the rat entopeduncular nucleus. Brain Struct. Funct..

[42] Chiken S (2015). Dopamine D1 Receptor-Mediated Transmission Maintains Information Flow Through the Cortico-Striato-Entopeduncular Direct Pathway to Release Movements. Cereb Cortex.

[43] Kliem MA (2007). Activation of nigral and pallidal dopamine D1-like receptors modulates basal ganglia outflow in monkeys. J Neurophysiol.

[44] de Jesus Aceves J (2011). Dopaminergic presynaptic modulation of nigral afferents: its role in the generation of recurrent bursting in substantia nigra pars reticulata neurons. Front Syst Neurosci.

[45] Atherton JF, Menard A, Urbain N, Bevan MD (2013). Short-term depression of external globus pallidus-subthalamic nucleus synaptic transmission and implications for patterning subthalamic activity. J Neurosci.

[46] Kita H, Kitai ST (1987). Efferent projections of the subthalamic nucleus in the rat: light and electron microscopic analysis with the PHA-L method. J Comp Neurol.

[47] Kita H, Kitai ST (1991). Intracellular study of rat globus pallidus neurons: membrane properties and responses to neostriatal, subthalamic and nigral stimulation. Brain Res.

[48] Awatramani GB, Turecek R, Trussell LO (2005). Staggered Development of GABAergic and Glycinergic Transmission in the MNTB. J Neurophysiol.

[49] Crins TTH, Rusu SI, Rodríguez-contreras A, Borst JGG (2011). Developmental Changes in Short-Term Plasticity at the Rat Calyx of Held. Synapse..

[50] Carter DA, Fibiger HC (1978). The projections of the entopeduncular nucleus and globus pallidus in rat as demonstrated by autoradiography and horseradish peroxidase histochemistry. J Comp Neurol.

[51] Penney JB, Young AB (1981). GABA as the pallidothalamic neurotransmitter: implications for basal ganglia function. Brain Res..

