# Supplementary material for: Short-term depression shapes information transmission in a constitutively active GABAergic synapse
Source: Sci Rep. 2019 Dec 2;9:18092. doi: 10.1038/s41598-019-54607-y (PMC6889381; doi:10.1038/s41598-019-54607-y)
Supplement: Supplementary file 1 — supplementary information [file 41598_2019_54607_MOESM1_ESM.pdf]

Short-term depression shapes information transmission in a constitutively active GABAergic synapse

Hagar Lavian[1] and Alon Korngreen

Supplementary figures

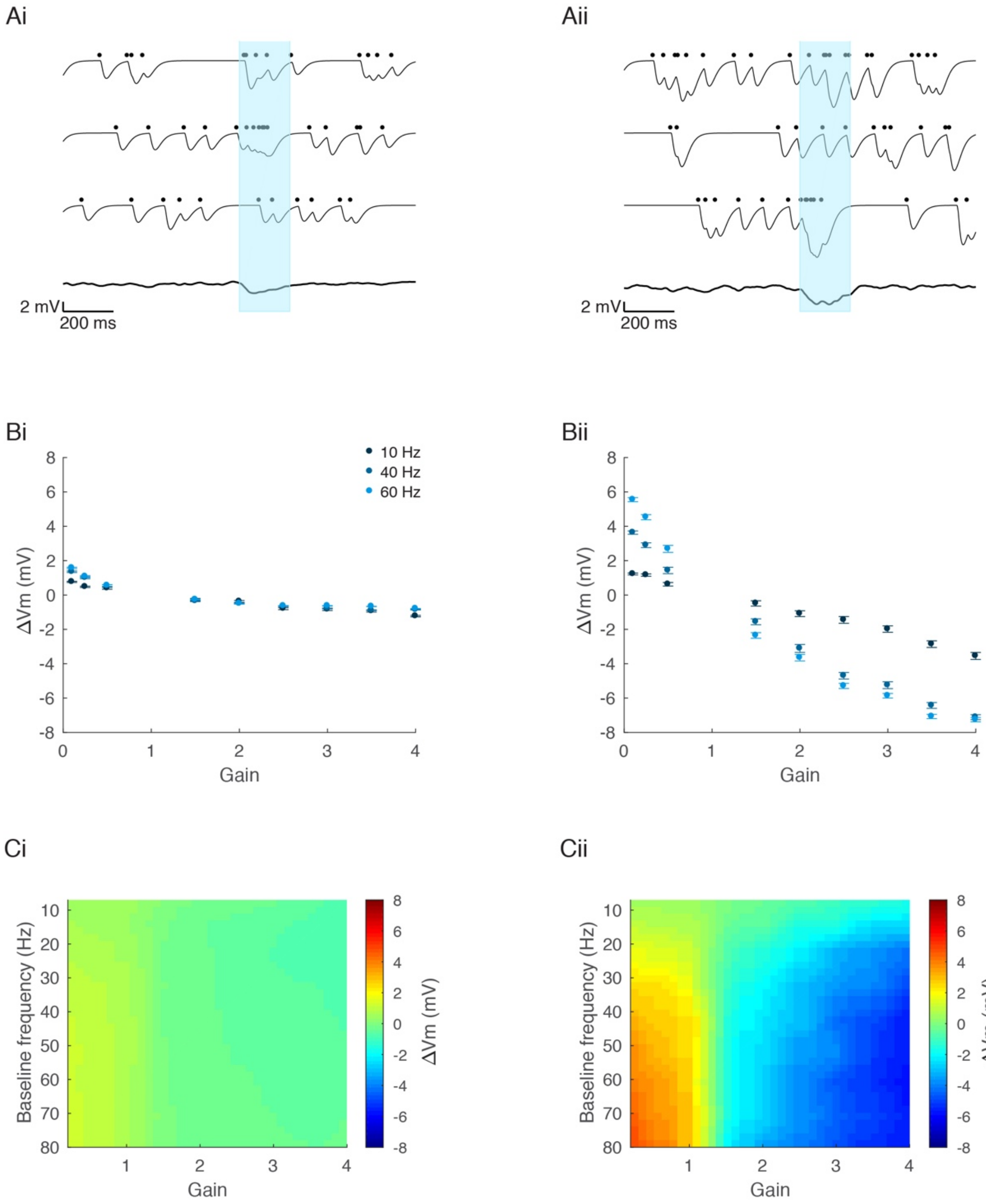

Figure S1: Gain control of the GP-EP synapse. A, simulated traces of GP evoked IPSPs induced by Poisson activation of a depressing synapse (i) and a non-plastic synapse (ii). The synapse was activated at 10 Hz, followed by a transient increase in activation rate to 30 Hz. B, predicted change in membrane potential as a function of the multiplication factor, calculated for baseline frequencies of 10, 40 and 60 Hz. C, predicted change in membrane potential as a function of the multiplication factor and the baseline firing rate.

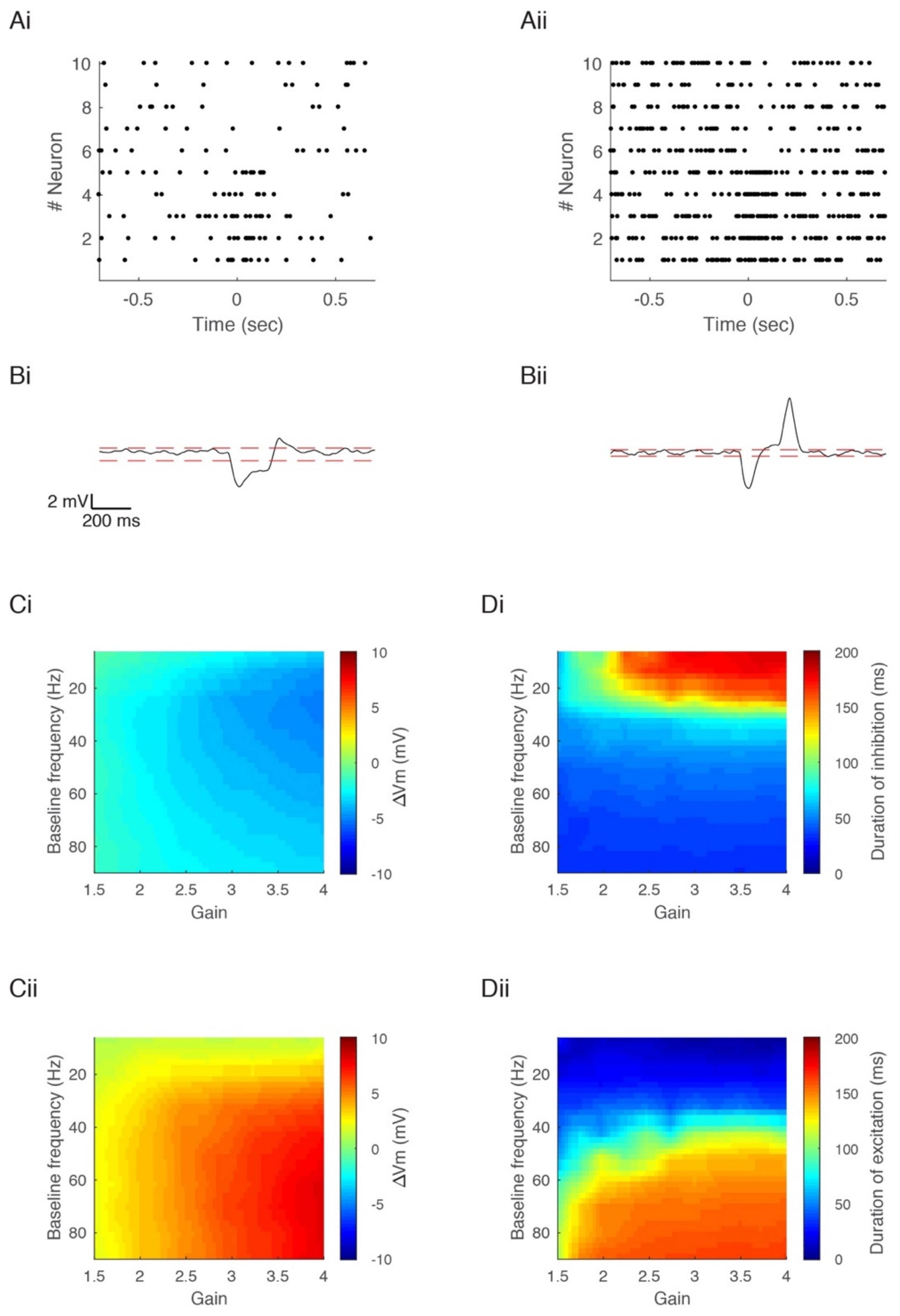

Figure S2: Different delayed firing of increasing or decreasing GP populations evoked short excitation of the EP. A, raster plot of the activity of 10 simulated GP neurons that change their firing rate for 200 ms. Half of the neurons increase their firing rate at t=-50 ms, and half of the neurons decrease their firing rate at t=0 ms. (i), example of low baseline firing rate. All 10 GP neurons have a baseline firing rate of 10 Hz and change their activity by 4/0.25. (ii), example of high baseline firing rate. All 10 GP neurons have a baseline firing rate of 40 Hz and change their activity by 4/0.25. B, predicted membrane potential of an EP neuron that receives input from 10 GP neurons in A. C, predicted change in membrane potential as a function of the

multiplication factor and the baseline firing rate during the inhibitory phase (i) or excitatory phase (ii). Calculated as the average change during t=0 ms and t=50 ms (i), and as the average change during t=150 ms and t=200 ms (ii). D, duration of the inhibitory phase (i) and excitatory phase (ii) induced by the change in presynaptic activity. The duration was defined as the time during which the postsynaptic membrane potential was significantly hyperpolarized (i, more negative than 2 SD below the mean membrane potential) or depolarized (ii).
